# Epigenetic Mechanisms in Aging: Extrinsic Factors and Gut Microbiome

**DOI:** 10.3390/genes15121599

**Published:** 2024-12-14

**Authors:** Alejandro Borrego-Ruiz, Juan J. Borrego

**Affiliations:** 1 Departamento de Psicología Social y de las Organizaciones, Universidad Nacional de Educación a Distancia (UNED), 28040 Madrid, Spain; a.borrego@psi.uned.es; 2 Departamento de Microbiología, Universidad de Málaga, 29071 Málaga, Spain

**Keywords:** epigenetics, aging, gut microbiome, microbial metabolites, neurodegenerative diseases, extrinsic factors

## Abstract

Background/Objectives: Aging is a natural physiological process involving biological and genetic pathways. Growing evidence suggests that alterations in the epigenome during aging result in transcriptional changes, which play a significant role in the onset of age-related diseases, including cancer, cardiovascular disease, diabetes, and neurodegenerative disorders. For this reason, the epigenetic alterations in aging and age-related diseases have been reviewed, and the major extrinsic factors influencing these epigenetic alterations have been identified. In addition, the role of the gut microbiome and its metabolites as epigenetic modifiers has been addressed. Results: Long-term exposure to extrinsic factors such as air pollution, diet, drug use, environmental chemicals, microbial infections, physical activity, radiation, and stress provoke epigenetic changes in the host through several endocrine and immune pathways, potentially accelerating the aging process. Diverse studies have reported that the gut microbiome plays a critical role in regulating brain cell functions through DNA methylation and histone modifications. The interaction between genes and the gut microbiome serves as a source of adaptive variation, contributing to phenotypic plasticity. However, the molecular mechanisms and signaling pathways driving this process are still not fully understood. Conclusions: Extrinsic factors are potential inducers of epigenetic alterations, which may have important implications for longevity. The gut microbiome serves as an epigenetic effector influencing host gene expression through histone and DNA modifications, while bidirectional interactions with the host and the underexplored roles of microbial metabolites and non-bacterial microorganisms such as fungi and viruses highlight the need for further research.

## 1. Introduction

Aging is a natural physiological process involving biological and genetic pathways and is linked to an increased susceptibility to several conditions, including cardiovascular, immune, metabolic, musculoskeletal, and neurodegenerative diseases [1]. Two primary theories have been proposed to explain the origin and progression of aging. The programmed or adaptive theory posits that a “genetic clock” dictates the timing of aging in an organism. In contrast, the damage or error theory attributes age-related phenomena to the diminished influence of natural selection as organisms grow older [2,3]. However, intrinsic and environmental factors, along with their interactions, have been suggested as pivotal mechanisms influencing the aging process in older individuals [4]. The World Health Organization has estimated that approximately 25% of the variability in longevity is attributable to genetic factors, while the remaining 75% is primarily influenced by interactions with environmental exposures [5]. According to Plagg and Zerbe [5], programmed aging is associated with age-related conditions such as cardiovascular diseases, neurodegeneration, and cancer. Nevertheless, understanding of the extent to which environmental factors impact aging remains incomplete. These factors include pathologies arising from specific environmental influences (e.g., air, buildings, climate, soil, social contexts, and water) that contribute to intrinsic mortality and premature death. In this respect, current studies on the regulation of aging should prioritize uncovering the roles of diverse endogenous and exogenous stressors, such as cellular senescence, disrupted intercellular communication, epigenetic modifications, genomic instability, impaired autophagy, mitochondrial dysfunction, and neuroinflammation [6]. Furthermore, growing evidence suggests that alterations during aging result in transcriptional changes, which play a significant role in the onset of age-related diseases, including cancer, cardiovascular disease, diabetes, and neurodegenerative disorders [7,8,9,10,11,12].

A simplistic definition of epigenetics includes changes in gene function that are heritable but do not implicate modifications to the DNA sequence [13]. A more widely accepted definition of this concept integrates the study of molecular markers that serve as reminders of previously encountered stimuli without causing permanent modifications in the genetic code [14]. The present knowledge of epigenetics involves various aspects related to shifts in the genetic code and phenotype through DNA methylation, histone modifications, and post-transcriptional changes of mRNA [13,15]. Significant epigenetic mechanisms observed in health and disease encompass covalent histone modifications such as acetylation/deacetylation, DNA methylation at cytosine residues, methylation, mRNA editing, and phosphorylation of histones [7,16,17].

The total genetic material of a host organism, together with that of its linked microorganisms, has led to the proposal of the concept of the hologenome [18]. According to the holobiont theory of evolution, natural selection can have an influence on the genome of the host, and on the metagenome of the microbiota, to form a structural entity [19]. The term holoepigenome has been employed by Stilling et al. [20] to describe the epigenetic characteristics and heritability of the gut microbiome (GM), and also by Oliveira [21] to determine the epigenetic processes involving the host and its symbionts. More recently, Pepke et al. [22] defined the holoepigenome as the entire collection of epigenetic alterations and their interrelations within the host genome. However, there are no studies that have examined both host and microbial epigenomes at the same time to clarify whether or not they collaborate as a cohesive holoepigenome for adaptation within the holobiont (a biological system that includes a host organism and its related microbiota).

Although it has been speculated that some transcription factors that affect host epigenetic processes may also influence GM epigenetics and gene regulation [23,24], it is currently believed that the GM may affect host epigenetic processes, resulting in negative effects [25]. Intrinsic and extrinsic factors, including age, diet, pharmacological influences, and toxic agents, trigger changes in the normal homeostasis of the GM, inducing a negative imbalance, termed dysbiosis, in the abundance and function of the microbiota as well as variations in microbial metabolites [26].

In the present narrative review, the epigenetic alterations in aging and age-related diseases have been explored, and the major extrinsic factors influencing these epigenetic alterations have been identified. In addition, the role of the GM and its metabolites as potential epigenetic modifiers has been addressed.

## 2. Epigenetic Mechanisms in Aging

Current knowledge has provided evidence for changes in chromosomal or mitochondrial DNA in aging and age-related dementia. For instance, DNA methylation markers are widely recognized as reliable indicators of biological age in animals, including humans, across various stages of life, even during developmental phases [27]. Moreover, the acceleration of age-dependent dementia and various aspects of aging, including life expectancy and longevity, is strongly correlated with epigenetic changes and also with extrinsic factors such as environmental influences [28,29]. In addition, many epigenetic changes have been identified with age in several health problems, such as metabolic diseases, cardiovascular diseases, carcinomas, and various neurodevelopmental processes [7,16,30].

DNA hypomethylation has been linked to aging, although hypermethylation also occurs at CpG islands, resulting in the formation of 5-methylcytosine [6,31]. The methylation levels of CpG sites serve as reliable indicators of biological age, referred to as epigenetic clocks that can estimate chronological age [32]. The second-generation epigenetic clocks, PhenoAge and GrimAge, incorporate additional factors into their predictions. PhenoAge includes morbidity and mortality, enabling predictions of 10- and 20-year mortality risks [33], while GrimAge offers a stronger predictive power for identifying clinical phenotypes [34]. Additionally, a recent study introduced a single-cell epigenetic age clock (scAge), which utilizes single-cell methylation data to determine epigenetic age [35].

In the context of neurological disorders, diverse studies have reported a significant link between alterations in DNA methylation and memory deficits stemming from age-related dementia [36]. For example, levels of 5-methylcytosine in the DNA of synaptic plasticity-related genes are substantially elevated in the prefrontal cortex of aged animals, resulting in transcriptional repression [17]. In addition, the memory-enhancing genes Arc and Egr1 exhibit reduced levels of DNA methylation-related enzymes in the hippocampus of older animals, with this reduction further influenced by learning processes [37,38]. Moreover, the age-associated decline in learning and memory may be attributed to a decrease in DNA methylation within the aging brain, which could be a key factor driving the reduction in adult neurogenesis observed in the aged hippocampus [39].

The major epigenetic mechanisms involved in age-related dementia encompass DNA methylation; histone modifications such as acetylation, methylation, and ubiquitination; mRNA modifications such as mRNA editing; and alternative splicing, as well as the involvement of non-coding RNAs in regulating gene expression [15,40].

### 2.1. DNA Methylation

DNA methylation is a chemical modification of a cytosine side chain, where a methyl group is added to the 5-carbon position of cytosine nucleoside residues, typically located in CpG-dense regions of the genome, through a covalent bond performed by DNA methyltransferases (DNMTs) [36,41,42]. The DNMTs catalyze this methylation in a nucleophilic reaction by transferring the methyl groups from the donor S-adenosylmethionine, thereby utilizing other micronutrients such as betaine, choline, folate, and vitamin B12 as additional essential cofactors [43,44].

DNA methylation is a crucial process that represses highly methylated genes, avoiding the transcriptional processes by RNA polymerase inaccessibility [27]. A high degree of DNA methylation throughout the genome causes embryonic mortality and several epigenetic changes of neuronal systems in mammals [45]. Neonatal death occurs due to the conditional deletion of the methyltransferase DNMT1 gene in neural precursor cells, leading to DNA hypomethylation within the central nervous system (CNS) and disrupting the neural regulation of respiratory function at birth [46]. On the other hand, motor coordination and learning remain intact by the targeted deletion of the DNMT1 gene within the brain, which leads to substantial DNA hypomethylation in both cortical and hippocampal neuronal areas [47]. In addition, DNA methylation is closely linked to mental health, including processes such as memory consolidation and overall cognition [48,49,50]. Inhibition of DNMT activity disrupts synaptic plasticity within the hippocampus and lateral amygdala, blocking the formation of hippocampus-regulated memory [51,52]. However, it remains to be determined whether DNA hypomethylation or hypermethylation is the major feature of aging and neurodegenerative diseases [53,54].

DNA methylation is mainly regulated by DNA methyltransferases (DNMT1, DNMT3A, and DNMT3B), with their expression levels being influenced by age. DNMT1 expression declines with age, leading to lower DNA methylation levels, while the expressions of DNMT3A and DNMT3B rise with age, contributing to de novo methylation of CpG islands in mammalian cells [55]. DNA methylation at gene promoters can result in gene silencing, whereas the removal of methyl groups from DNA is facilitated by ten-eleven translocation (TET) enzymes [56]. In clinical studies, mutations in TET2 or DNMT3A have been shown to elevate the expression of pro-inflammatory cytokines, contributing to chronic inflammation in elderly patients, which is linked to the development of cardiovascular diseases [57].

Aging is the major and most-studied risk factor for Alzheimer’s disease (AD) [58], and as age advances neurodegeneration escalates, which is age-associated DNA methylation through epigenetic dysregulation in AD and linked dementia [59]. Several studies agree that the level of DNA methylation of the whole genome is lower in AD patients than in healthy individuals [60,61]. However, other studies contradict these findings and suggest that the genome in frontal cortex neurons is globally hypermethylated in AD patients [62,63]. Shireby et al. [64] have demonstrated that diverse DNA methylation changes associated with AD pathology in the bulk cortex are due to alterations in non-neuronal cells such as oligodendrocytes, suggesting that changes in DNA methylation in astroglial cells could influence several types of neurodegenerative processes through changes in their DNA methylation and other epigenetic pathways. In addition, age-related epigenetic modifications probably are likely driving functional and structural modifications that increase susceptibility to neurodegenerative disorders and result in a progressive cognitive deterioration [15]. For example, age-related demethylation in the promoter regions of the amyloid precursor protein (APP) gene due to aging leads to the concentration of Aβ peptides in the brain, ultimately resulting in dementia [61,65].

Other functions related to the brain, including brain development, the differentiation of brain cell types, plasticity, memory, and learning processes, have also been associated with DNA methylation [66]. DNA methylation resulting from MeCP2 gene mutations is a common factor in neurodevelopmental disorders such as Rett syndrome, Autism Spectrum Disorder (ASD), and Fragile X syndrome [67,68]. Furthermore, neuronal hyperexcitability and epileptogenesis in subjects who have been diagnosed with temporal lobe epileptic seizures are linked to the hypermethylation of the monocarboxylate transporter 4 (MCT4) genes, particularly in astrocytes [69].

### 2.2. Histone Modifications

Chromatin structure is crucial for regulating gene expression and mediating gene–environment interactions through epigenomic adjustment [70,71]. Given that gene expression underpins cognitive stability and synaptic plasticity, the modulation of chromatin structure has been referred to as chromatin plasticity [72]. Histone proteins are fundamental in preserving the three-dimensional chromatin configuration and in enabling interactions between transcription factors and gene promoters [36]. The addition of functional groups to histone tails, known as chromatin remodeling/modification, provides two primary purposes: (i) acting as recruitment signals for non-histone proteins that have a role in either activating or repressing gene expression [36], and (ii) loosening chromatin structure by altering nucleosomal organization, disrupting nucleosomal contacts, and reducing the interactions between histone tails and DNA [73].

Histone acetylation by histone acetyltransferases (HATs) has been implicated in promoting learning and memory formation as well as cognitive decline [74,75,76]. In addition, histone acetylation and deacetylation are pivotal processes in controlling gene expression, as they modulate DNA accessibility to RNA polymerases [77]. In older adults with dementia, histone acetylation undergoes notable alterations and is frequently linked to cognitive decline, including challenges in forming new memories and forgetfulness [78,79]. In contrast to HATs, histone deacetylases (HDACs) target lysine residues on histone and non-histone DNA-binding proteins, removing acetyl groups from these molecules [80]. In addition, they regulate transcription via the interaction with diverse transcriptional factors, thereby influencing cellular processes such as cell proliferation and differentiation through the enhancement of gene expression [81]. Evidence suggests that HDACs are essential for cognition-enhancing processes, including memory consolidation and neurogenesis, as these enzymes exhibit alterations in dementia and in some neurodegenerative disorders [78,82]. Research indicates that substantial shifts in gene expression take place in brain regions associated with advanced cognitive functions, such as the hippocampus and the prefrontal cortex, particularly during and after the acquisition of new information or the retrieval of stored memories [83]. Several environmental and lifestyle factors are essential regulators of epigenetic changes, and histone acetylation in the hippocampus in particular has been closely associated with age-related changes in gene expression [59,71].

Histone methylation at N-terminal arginine or lysine residues regulates gene expression, with its disruption linked to the development of various pathological conditions [72,84]. This chemical modification is performed by enzymes histone methyltransferases (HMTs) [85], resulting in methylation at arginine or lysine residues, which causes transcriptional activation or repression of modified genes [72,85]. Numerous studies have highlighted that alterations in the levels of histone methylation-associated enzymes on a global scale play a pivotal role in the onset of dementia and other neurodegenerative diseases. These changes are accompanied by significant shifts in the dynamics of gene expression, particularly in genes linked to aging and cognitive function [84,86,87].

Other histone modifications consist of the aggregation of a phosphate or ubiquitin group to the histone tails of the nucleosome, which significantly contributes to the development of dementia [86,88,89]. In addition to acetylation, many other types of acylation to histone lysines have been identified, including crotonylation, propionylation, succinylation, and malonylation, [90], although the role of these histone acylations in neuroscience remains elusive [91].

Post-translational modifications of histones play a crucial role in regulating gene expression, either activating or silencing it. The most prevalent modifications observed during aging are acetylation and methylation, although phosphorylation, ubiquitination, and ADP ribosylation have also been documented [6]. Both in vivo and in vitro studies have shown alterations in the levels of several histone modifications, including H3K9me3 (trimethylation at lysine 9 of the histone H3 protein), H4K20me3 (trimethylation at lysine 20 of the histone H4 protein), H3K27me3 (trimethylation at lysine 27 of histone H3), and H3K9ac (acetylation at lysine 9 of histone H3), all of which are directly linked to aging and longevity [6]. H3K4me3 (trimethylation at lysine 4 of histone H3) is particularly influential in regulating aging and lifespan by modulating the expression of genes associated with aging. In contrast, H3K27me3 is linked to gene silencing and the formation of compacted heterochromatin, while H3K36me3 (trimethylation at lysine 36 of histone H3) and H3K9me3 are linked to a reduced lifespan [92].

### 2.3. Post-Transcriptional Modifications of mRNA

mRNA processing by mechanisms such as intron removal, alternative exon ligation, and the modification of mRNA base sequences, is crucial for epigenetic regulation. Modifications of mRNAs by polyadenylation and alternative splicing, together with the regulation of gene expression by long non-coding RNA molecules and RNA methylation, represent an additional layer of epigenetic regulation, comparable to histone modifications or DNA methylation [93,94,95]. These mRNAs and non-coding RNA-mediated epigenetic changes significantly increase the intricacy of the neuronal transcriptome [96] and may contribute to the development of several diseases, such as age-related dementia, cancer, cardiovascular disease, and neurodegeneration [9,15,96,97,98,99,100].

mRNA editing implicates modifications to the base sequence, primarily through the deamination of adenine to inosine (mediated by the enzyme adenosine deaminase, ADARs) or cytosine to inosine or uracil, facilitated by APOBECs (single-stranded DNA cytosine-uracil deaminase enzymes) [101,102]. This mRNA editing process is pivotal in the onset and development of cognitive impairment and neurodegenerative diseases [103,104]. mRNA editing can impact gene expression by altering splice sites, introducing coding changes, modifying microRNA (miRNA)-binding sites, or influencing other RNA processing mechanisms, all of which play a role in dementia and neurodegeneration [96,105].

Several lines of evidence indicate that non-coding RNAs (ncRNAs) have a significant impact on gene expression, influencing epigenetic, transcriptional, and post-transcriptional regulation, thereby playing an important role in age-related dementia and neurodegeneration [6]. Numerous ncRNAs are involved in these disorders, including miRNAs, circular RNAs (circRNAs), and long non-coding RNAs (lncRNAs), which work together in a synergistic manner to regulate gene expression under both healthy and pathological conditions [106,107,108]. Small ncRNAs, such as miRNAs, are key regulators of gene expression and act by recognizing specific nucleotide sequences, a process known as RNA silencing. They bind to the 3′ untranslated region (3′UTR) of target mRNAs, leading to mRNA degradation or translation inhibition, thereby influencing gene expression [109]. For example, miRNAs modulate the expression of genes implicated in Aβ metabolism, such as the target microtubule-associated protein tau (*MAPT* gene), amyloid precursor protein (*APP* gene), and β-site APP-cleaving enzyme 1 (*BACE1* gene), and they also regulate the alternative splicing of tau and APP [110,111]. In addition, many of these miRNAs, such as miR-146a-5p, are significantly upregulated in microglial cells within the aging brain, where they participate in neuroinflammation-driven neurodegeneration and in cognitive deterioration [112,113]. Moreover, other miRNAs, like miRNA-132 and miRNA-212, were downregulated in AD patients with mild cognitive impairment (MCI), especially within the frontal cortex area [114]. Additionally, circRNAs, a type of single-stranded and exceptionally stable ncRNA, are also critical in the development of neurodegenerative diseases and of age-related dementias, as they exhibit elevated expression levels in brain tissues and in inflammatory neuropathy [115]. Recently, lncRNAs have garnered considerable attention for their pivotal role in the pathogenesis of neurodegenerative diseases. Nevertheless, there are substantial gaps in knowledge regarding their biological functions, their molecular mechanisms, and their potential relevance in these diseases [116].

Alternative splicing of nascent and pre-mRNAs is a key post-transcriptional regulatory mechanism that influences gene expression dynamics, thereby promoting proteomic and functional diversity [117]. This process, which occurs within pre-mRNA transcribed from a single gene, enables the production of multiple mRNAs that encode various proteins with distinct structures, cellular roles, and subcellular localizations [118,119]. The spliceosome, a complex comprising small nuclear RNAs (snRNAs) U1, U2, U4, U5, and U6, along with other associated proteins, mediates alternative splicing [120]. Known mechanisms driving alternative splicing include exon shuffling, constitutive exon splicing, and the exonization of mobile transposable elements [117,121]. The role of alternative splicing is well-documented in neurodegeneration, age-related cognitive decline, and dementia [40,122]. It contributes to these processes through splicing variants of APP, apolipoproteins (APOE3 and APOE4), and tau proteins [123,124]. Aberrant splicing in aging and related diseases could lead to protein alterations and misfolding, although it is not known whether these splicing anomalies are causal or merely a consequence of the aging phenotype.

Finally, the maturation of mRNA from pre-mRNA implicates a pivotal process in which a complex of proteins cleaves the 3′ end of the mRNA and attaches adenine residues, forming a poly(A) tail. Polyadenylation occurs at various sites within a single mRNA molecule, depending on the availability of polyadenylation signals, resulting in the production of distinct mRNA isoforms with varying 3′ UTR lengths. This phenomenon is referred to as alternative polyadenylation (APA), a post-transcriptional mechanism that is critical for transcriptome diversification [125,126]. Altered APA patterns have been observed in several neurodegenerative diseases [127,128], where the changes in APA profiles are restricted to a limited set of genes, with only a few genes affected, primarily those linked to protein turnover and mitochondrial function, such as ubiquitin protein ligase E3 component N-recognin 1 (UBR1) and oxoglutarate dehydrogenase L (OGDHL) [129].

## 3. Extrinsic Factors in Epigenetic Aging

The hallmarks of aging include three current major categories of age-associated mechanisms that are central to aging processes, namely primary, antagonistic, and integrative features [130]. Primary features involve factors that undermine cellular integrity, such as genomic instability, telomere attrition, epigenetic modifications, and the loss of proteostasis. These contribute to cellular senescence, oncogenesis, and various age-associated diseases [130,131,132]. Antagonistic features include disturbances in metabolic pathways, cellular senescence, and mitochondrial dysfunction, leading to diminished tissue elasticity and the activation of inflammatory pathways [133,134,135,136]. Integrative features affect cellular homeostasis, including the depletion of stem cells and dysregulated cellular signaling [130,137,138].

More recently, twelve distinct hallmarks of aging have been identified, including epigenetic changes, genomic instability, telomere shortening, loss of proteostasis, impaired macroautophagy, disrupted nutrient sensing, mitochondrial dysfunction, chronic inflammation, cellular senescence, altered intercellular communication, stem cell depletion, and dysbiosis of the GM [139,140]. Therefore, phenotypic indicators of aging are frequently observed in the elderly, particularly among individuals that present frailty and neurocognitive deficits. Nevertheless, defining a clear epigenetic profile for either elderly or youthful individuals remains challenging. Many efforts have been made to establish the significance of epigenetic mechanisms in aging, with a particular focus on DNA methylation [6]. It is proposed that accumulated environmental stress over a lifetime disrupts epigenetic patterns, leading to transcriptional noise and contributing to the aging phenotype [141]. Although the loss of epigenetic signatures plays a crucial role in aging, recent studies have demonstrated that these changes can be reversed in animal models, offering the potential for reversing aging processes [142].

However, factors such as diet, drug use, environmental chemicals, and physical activity can induce epigenetic alterations via the endocrine and immune pathways, which may possess important implications regarding longevity [143,144,145]. The influence of these extrinsic and/or environmental factors has been determined in twin studies, some of which reported that 70–80% of epigenetic variance is attributable to external factors [146,147]. In this sense, epigenetic variability is more pronounced in older twin pairs, consistent with the stress accumulation hypothesis [147]. DNA methylation mechanisms often lose their original hypo- or hyper-methylated states with age, gradually becoming more balanced [148,149]. The significance of epigenetics in the aging process is further highlighted by studies on epigenetic rejuvenation, which have demonstrated that altering DNA methylation patterns can promote tissue regeneration and inhibit cellular senescence [150]. However, specific epigenetic changes induced by environmental factors may accelerate the aging process.

Epigenetic clocks utilize a variety of cytosine-phosphate-guanine (CpG) sites across the genome to estimate epigenetic age. Various clocks have been developed, but only four are widely considered as reliable indicators of mortality risk [151]. According to Levine et al. [33], the Hannum and Horvath clocks were created by identifying specific sets of CpGs whose DNA methylation levels change with age, while the PhenoAge clock is based on a separate biological age measure, incorporating age and nine clinical biomarkers. The GrimAge clock is a composite marker, integrating seven sets of CpGs, each linked to the concentration of a distinct plasma protein. Epigenetic age acceleration, which reflects the difference between biological and chronological age, can be assessed using the four epigenetic clocks mentioned above. This acceleration has been shown to correlate with a number of lifestyle and health-associated factors [152,153].

### 3.1. Negative Influence of Chemical Compounds on Epigenetic Aging

Epigenetic toxicity is a phenomenon in which chemicals and xenobiotics affect the epigenome and exert detrimental impacts on living organisms, potentially accounting for the long-term effects of chemicals and xenobiotics, as well as increased susceptibility to disease due to these environmental influences [154]. Recalcitrant chemicals and xenobiotics are abundant in our environment, and organisms are continuously, knowingly, or naively exposed to these compounds. These substances exert pleiotropic effects ranging from curable to fatal conditions and are therefore a health concern. Depending on the type, dose, and duration of exposure, xenobiotics alter the genetic and epigenetic status of an organism, resulting in dysregulated gene regulation and expression and in phenotypic abnormalities. Table 1 describes the major chemicals and xenobiotics that induce changes in the nervous system.

The epigenetic alterations related to smoking have been associated with changes in gene expression, including the activation of senescence genes [183,184]. In particular, smoking accelerates the epigenetic age of airway epithelium and lung tissue by approximately four to five years [185], and smoking during adolescence causes irreversible damage that can be identified later in life [186]. Early cessation of smoking can restore the normal rate of airway epithelial aging, but the lungs of former smokers retain the imprint of accelerated aging [187].

Individuals with alcohol use disorder (AUD) exhibit distinct methylation patterns compared to healthy controls [188], indicating that epigenetic clocks could help track how changes in methylation in these individuals correspond to alterations in DNA methylation age. This could provide insights into the shared molecular mechanisms linking alcoholism and aging. The negative impact of AUD on epigenetic age includes harmful effects on the cardiovascular and neural systems [189], as well as the acceleration of biological aging in regular drinkers [190]. Notably, the degree of accelerated aging has been linked to polymorphisms in the *APOL2* gene, potentially affecting its expression in the hippocampus. The *APOL2* gene is also implicated in addiction and schizophrenia, suggesting that accelerated epigenetic aging might serve as a marker for certain mental health and substance abuse disorders [191]. The specific type of alcohol consumed seems to play a significant role in alcohol-related epigenetic aging. While consumption of spirits, beer, and liquors is associated with an increase in biological age, wine consumption does not show the same correlation, likely due to its high polyphenol content [192].

Exposure to various drugs results in epigenetic and phenotypic alterations across cellular, circuit, and behavioral dimensions. Drugs with epigenetic effects can be categorized into two groups: (i) those that directly interact with the epigenetic mechanisms, primarily targeting epigenetic adders and removers, and (ii) those that, through their effects on non-epigenetic targets (e.g., receptors), trigger intracellular signaling pathways, transcriptional changes, or both, ultimately leading to modifications of the epigenome [193].

Machado et al. [194] concluded that there is evidence for exocannabinoid-induced epigenetic modifications that modulate addictive, anxious-depressive, and psychotic behavioral phenotypes. Several investigations have examined methylation at certain genes, finding that cannabis exposure was linked to decreased methylation at several genes, such as aryl hydrocarbon receptor repressor (*AHRR*), *DNMT1*, dopamine D2 receptor (*DRD2*), catechol-O-methyltransferase (*COMT*), DLGAP2, arginase 1 (*Arg1*), signal transducer and activator of transcription 3 (*STAT3*), methylguanine methyltransferase (*MGMT*), and proenkephalin (*PENK*), while hypermethylation was found in the gene DNA methyltransferases Dnmt3a and Dnmt3b (*DNMT3a/b*), neural cell adhesion molecule 1 (*NCAM1*), and *AKT1* kinase genes. However, further research will require the standardization of drug administration and dosage protocols, as well as the development of a tailored gene pool to evaluate the potential of these genes as biomarkers for neurodegenerative and neuropsychiatric conditions.

In addition, Vaillancourt et al. [195] established a causal relationship between cocaine exposure and the methylation of the *IRX2* gene, CTCF protein binding, three-dimensional chromatin interactions, and subsequent gene expression. In this regard, the hypomethylation of *IRX2* plays a role in the development and persistence of cocaine dependence by inducing alterations in the 3D chromatin structure within the caudate nucleus.

Current evidence suggests that opioids contribute to increased levels of permissive histone acetylation, reduced repressive histone methylation, and alterations in DNA methylation patterns, as well as non-coding RNA expression, across the reward circuitry of the brain [196,197]. A preclinical study indicates that even a single exposure to heroin can lead to differential gene expression in the hippocampus of non-human primates of varying ages. Specifically, genes related to the mitogen-activated protein kinase (*MAPK*) signaling pathway (*STMN1*, *FGF14*, and *MAPT*), γ-aminobutyric acid (GABA)-ergic synapses (*GABBR2* and *GAD1*), neurotrophin signaling (*NTRK1* and *NGFR*), autophagy (*ATG5*), and dopaminergic synapses (*AKT1*) in the striatum were found to be differentially expressed in heroin-treated animals compared to controls [198].

### 3.2. Diet and Epigenetic Aging

Dietary factors that affect epigenetic aging have been extensively studied to understand if they can be used as potential geroprotective agents [199]. Gensous et al. [200] found that reducing calorie intake can prevent the age-related methylome in model organisms, revealing that a caloric restriction (CR) diet may exert an impact on the epigenetic signatures of aging. In preclinical studies, it has been reported that short-term CR intake induces a remodeling of the DNA methylation of several genes implicated in diabetes, inflammation, and cardiovascular health-associated pathways [201], whereas long-term CR delays the onset of age-related pathologies and improves subsistence in animals, including humans [202,203].

To date, the influence of CR in humans has not been fully determined, but efforts have been made to extrapolate findings from primate studies [204,205], and several studies have been performed recently. Fitzgerald et al. [206] conducted an eight-week pilot clinical trial combining CR with exercise, nutritional supplements, and counseling in 43 older adult men. The regimen resulted in an epigenetic age reduction of 2–3 years using Horvath’s clock. Similarly, in another study called CALERIE, 128 subjects had their caloric intake diminished by 25% and were followed over 2 years [207]. A small but statistically significant alteration in the rate of aging was observed, although it did not result in substantial shifts regarding biological age estimates as determined by diverse DNA methylation clocks, such as GrimAge and PhenoAge. The 2–3% slowing of aging found in the CALERIE trial was regarded as corresponding to a 10–15% decrease in mortality risk.

Various other dietary patterns have also been assessed for their potential to mitigate epigenetic aging, including the Dietary Approaches to Stop Hypertension (DASH), Healthy Eating Index-2015 (HEI), Alternative Healthy Eating Index (aHEI-2010), and Alternative Mediterranean (aMED) diet. These indices gauge the healthfulness of a diet by assigning points based on dietary composition, with higher scores awarded to individuals who consume greater amounts of vegetables, fruits, proteins, polyunsaturated fats, and omega-3 fatty acids, while those who consume less alcohol, sugar, and processed foods receive higher scores [208,209,210,211]. While assessing the influence of these diets on epigenetic age in 2694 adult women, Kresovich et al. [212] found significant associations with dietary index adherence using the PhenoAge and GrimAge epigenetic clocks. Interestingly, the beneficial effect of a healthy diet was greater in subjects with low levels of physical activity, although women with more than 2.5 h of activity per week did not show this aging slowdown at all. Therefore, the authors proposed that diet and exercise influence the same epigenetic pathways, as indicated by PhenoAge, suggesting that their effects may not be cumulative.

In another recent study, with a case series of six women who followed a methylation-supportive diet and lifestyle program (which included diet, exercise, sleep, and relaxation guidance, as well as supplemental probiotics, phytonutrients, and nutritional coaching) designed to affect DNA methylation and measures of biological aging, Fitzgerald et al. [213] found that participants’ epigenetic age was reduced by an average of 4.6 years old (range 0–11 years) after 8 weeks of treatment.

It is interesting to note the influence of several components of a particular diet. In this sense, the intake of a diet rich in fruits and vegetables was linked to a deceleration of the aging process [153]. Polyphenols have recently received much attention from the anti-aging community, with catechol-dominant polyphenols shown to inhibit enzyme activity and also to activate epigenetically silenced genes [214]. In addition, other dietary polyphenols, such as curcumin, quercetin, or pterostilbene, show effects against aging due to their influence on the epigenome modulated by their interplay with DNMTs, miRNAs, and histone modifiers [215,216,217]. Additional beneficial effects of polyphenols are provoked by their interactions with sirtuins, which are proteins implicated in cellular senescence, circadian rhythm regulation, DNA re-establishment processes, and stress response [215]. Polyphenol epigallocatechin-3-gallate, found in green tea, attenuates age-related systemic inflammation, oxidative stress, and apoptosis and extends the lifespan of rats by 8–12 weeks [218,219], while resveratrol, found in berries, grapes, and peanuts, shows antimicrobial properties [220] and offers various health benefits, such as anti-obesity, antidiabetic, antitumor, antioxidant, cardioprotective, neuroprotective, and anti-aging effects, and it also increases sirtuin expression [221].

Different foods, including coffee, garlic, parsley, and nuts, have been suggested to influence epigenetic aging due to their high levels of phytochemicals [222,223,224,225]. However, although phytochemicals have demonstrated potential in mitigating the progression of age-associated brain diseases, further research is needed to determine their efficacy, by themselves or in combination, within the epigenetics of nutrition. In short, the idea of an epigenetic rejuvenation through the intake of a certain diet has been proposed [226], but it is not fully established because the rejuvenating effects of certain ingredients or dietary patterns have not been adequately assessed, primarily due to the presence of numerous confounding factors.

The metabolites generated by the gut microbial degradation of undigested fibers can produce epigenetic changes by repressing gene expression of the methylated region by repressing or promoting gene expression according to the type of histone modification, and by diminishing the translation efficiency of mRNAs or promoting their degradation [227]. Phenolic compounds can modulate gene expression through the regulation of epigenetic mechanisms. These compounds are able to activate HDACs, activate or inhibit HATs, activate sirtuins, and inhibit DNMTs [228], and they are also associated with the regulation of miRNA expression [229]. It has been suggested that there is an association between epigenetics, CR, and biological longevity [230]. Results from preclinical studies have shown that epigenetically predicted age is significantly lower in animals under CR conditions [231,232]. Table 2 shows the influence of dietary components on the GM, epigenetic changes, and related-biological activities.

### 3.3. Physical Activity and Epigenetic Aging

Large-scale studies have revealed that individuals who perform regular exercise exhibit a lower mortality rate compared to the least active people within the same age group [233]. Thus, physical activity can be considered one of the key factors in human longevity. The obvious advantages of training can be viewed as a slowing of aging measured by epigenetics aging clocks. Several studies have investigated whether or not the epigenetic modifications associated with physical activity are similar to those linked to a slower aging process. For example, individuals engaged in lifelong physical exercise present reduced DNA methylation levels at gene promoters in muscle tissue [31,234]. In contrast, increased DNA methylation levels at gene promoters have been observed in adipose tissue following physical activity [235,236]. These contrasting findings may stem from tissues-specific responses, differences in experimental designs, the non-uniform effects of exercise on the epigenome across various tissues, and the limited exploration of the longevity of epigenetic changes induced by physical activity.

The impact of physical activity and weight control on mitigating various indicators of aging has been extensively documented through longitudinal research [237]. Although several studies emphasize the potentially positive DNA methylation shifts triggered by exercise, epigenetic aging clocks often fail to reflect these changes as a deceleration of aging [34,238,239].

Physical activity has been linked to the rate of aging, with epigenetic aging also showing a notable association with body mass index (BMI). Studies involving twins and obese individuals have revealed that a 10-unit increase in BMI correlates with an epigenetic age increase of 1–3 years in blood, liver, and adipose tissue [240,241]. Although both BMI and exercise influence epigenetic age, the connection between adiposity and epigenetic aging is generally not confounded by physical training [242]. Therefore, BMI and exercise should not be considered interchangeable when assessing fitness in terms of epigenetic aging.

### 3.4. Psychological Stress and Epigenetic Aging

Recent findings indicate that psychological stress can expedite epigenetic aging [243]. It has been recognized that psychological stress impacts human longevity at the molecular level by promoting oxidative processes and telomere homeostasis [244]. In aging, it has also been demonstrated that accumulated stress over a lifetime can imprint lasting changes on the epigenome. In this respect, cumulative stress over a lifetime may accelerate epigenetic aging, potentially driven by glucocorticoid-induced changes in the epigenome, whereas no significant association was found with childhood maltreatment or current stress alone [245]. Thus, both stress and glucocorticoids can induce not only acute changes in gene transcription but also epigenetic modifications, most notably changes in DNA methylation [246,247].

Consequently, lifetime stress may contribute to a biological age difference of up to 3.6 years between individuals [248]. Other findings suggest that traumatic stress experienced in childhood and adulthood is associated with aging rate (approximately 2 years epigenetically older) in later life [249,250]. In addition, being a direct victim of violence is linked to increased epigenetic age in children [251]. As a result, interventions designed to reduce stress, such as mindfulness-based stress reduction, may positively influence markers of epigenetic aging. For instance, meditation has been found to reduce epigenetic age in a cumulative manner, with each year of practice corresponding to a 0.24-year decrease in biological age [252].

In an animal model, alpha male baboons were found to be epigenetically one year older than their chronological age, with ascending male dominance hierarchy linked to accelerated aging [253]. However, no significant association was found between dominance rank and epigenetic age in female baboons. The authors proposed that the heightened competitiveness at the top of the hierarchy, along with the corresponding changes in glucocorticoid levels, may drive the accelerated aging observed in male baboons [254].

Social stress is thought to become biologically encoded via the epigenetic modifications of genes such as the glucocorticoid receptor (*GCR*), corticotropin-releasing hormone (*CRH*), and *FKBP5* [255,256]. Evidence suggests that the absence of close relationships, particularly with family members and friends, is associated with accelerated epigenetic aging in older adulthood. This provides one potential mechanism through which social relationships might influence the risk of age-related decline and disease [257].

Chronic stress has been linked to negative long-term health outcomes, raising the possibility that stress contributes to accelerated aging. In a study, Harvanek et al. [258] investigated whether or not resilience factors could affect stress-associated biological age acceleration. They demonstrated that cumulative stress was associated with epigenetic aging in a healthy population, and these associations were influenced by biobehavioral resilience factors. Thus, repeated or prolonged exposure to stressful life experiences may accelerate the rate at which organisms ages.

The mechanisms through which chronic psychosocial stress influences biological aging pathways likely involve multiple physiological and molecular networks. Chronic psychosocial stress activates neuroendocrine responses via the sympathetic nervous system (SNS) and the hypothalamic-pituitary-adrenal (HPA) axis. Activation of the SNS results in the release of norepinephrine from neuronal axon terminals that innervate tissue in immune and other organs, such as the thymus and spleen, as well as the release of both norepinephrine and epinephrine into the circulation system. The HPA axis regulates the release of glucocorticoids (cortisol in humans) from the adrenal cortex into the bloodstream, allowing them to circulate throughout the body. These neuroendocrine mediators affect numerous physiological processes that may play critical roles in biological aging [259].

The free radical theory of aging posits that aging results from accumulated damage caused by reactive oxygen species (ROS), which lead to cellular senescence and oxidative DNA damage [260]. Epigenetic regulation is implicated in endogenous oxidative stress-related aging processes, driven by the hyperactive production of intracellular ROS and the impairment of antioxidant defense systems.

Recent findings link low socioeconomic status to epigenetic age acceleration, as measured by DNA methylation markers [261,262]. In addition, Freni-Sterrantino et al. [263] explored the associations between job strain, effort–reward imbalance, and work characteristics and five biomarkers of epigenetic aging (Hannum, Horvath, PhenoAge, GrimAge, and DunedinPoAm). The results they obtained indicated that job-related stress variables were associated with small magnitudes of age acceleration, within a range of 2 years, compared to the effects of non-communicable disease risk factors.

### 3.5. Other Extrinsic Factors and Epigenetic Aging

Long-term exposure to ambient air pollution is linked to a variety of adverse age-related outcomes, including aging and epigenetic alterations [264]. Prolonged air pollution exposure has also been associated with shortened telomeres, indicating an accelerated aging process [265]. However, a more recent study failed to replicate these associations [266]. Ward-Caviness et al. [267] reported that exposure to ambient particulate matter is associated with the epigenetic biomarkers of aging. Exposure to mono-nitrogen oxides and black carbon (BC) was associated with accelerated epigenetic aging in females. In contrast, in males, the direction of the association for these exposures was opposite, although not statistically significant. A similar observation was noted for particulate matter, where men exhibited significant inverse associations with epigenetic age acceleration and intrinsic epigenetic age acceleration, while women showed positive, but non-significant, associations. Additionally, BC was inversely associated with telomere-length-based age acceleration in men. More recently, Koenigsberg et al. [268] found that higher levels of particulate matter and NO_2_ were associated with increased epigenetic aging among Black women, but not among non-Hispanic White (NHW) women. These authors also identified 19 differentially methylated CpG sites associated with pollutants in Black participants and one site in NHW participants.

Another extrinsic factor is exposure to radiation. Ionizing radiation is typically used to create models of accelerated aging, as the processes of aging and radiation injury share common mechanisms [269]. Increasing evidence shows a close relationship between human radiation sensitivity and age-related health effects, with ionizing radiation accelerating many of the normal aging processes. Individuals exposed to ionizing radiation are most radiosensitive at early ages, with sensitivity to radiation decreasing until maturity, but then increasing again in older ages [269,270]. Ruprecht et al. [271] explored the epigenetic landscape of radiation exposure and its effects on aging processes. The authors identified 17 methylated genes related to oxidative stress, mechanisms of cell development, growth and death, immune response, and metabolism/energy, with no direct links to mitochondrial dysfunction. Interestingly, radiation exposure induces changes in a gene involved in hematopoietic cell differentiation, *RBM47*, which is linked to one of the hallmarks of aging: stem cell exhaustion. Thus, these findings provide tentative support for a radiation-aging association.

The patterns of gene expression shaped by molecular epigenetic marks established during development influence human vulnerability to various diseases, including viral infections. These infections involve alterations in chromatin structure or nucleic acid chemical properties without directly modifying the genetic code, unlike mutations that disrupt genetic material [272]. Beyond their physiological roles, deregulated epigenetic processes are increasingly recognized as contributors to severe diseases, including microbial infections [273]. Several studies suggest that infection-induced changes in the cellular epigenome are linked to pathological processes in both acute and chronic infections. Persistent viruses, chronically infecting bacteria, and protozoa exploit these permanent cellular changes, which include cell cycle regulation, control of apoptosis, and immune system suppression. This phenomenon is observed in several virus families [274], intracellular bacteria (e.g., *Escherichia coli*, chlamydia, mycobacteria, *Legionella*, and *Helicobacter pylori*) [275], and parasites such as *Cryptosporium*, *Toxoplasma*, and *Theileria* [276].

Interestingly, viruses belonging to the *Coronaviridae* and *Orthomyxoviridae* families are incapable of directly altering the genetic sequence of the host, but they can modify its epigenome. Recent research has focused on how viruses exploit aspects of the epigenetic machinery to facilitate infection, spread, and persistence [277]. It has been shown that several pathogenic viruses antagonize the immune system by employing various epigenetic mechanisms, such as SARS CoV-2, which influences the epigenomic markers of immune cells in the host, thereby modulating the immune response and affecting the outcomes of the infection [278]; the H3N2 influenza A virus, which inhibits the initiation of the innate immune response of the host by interfering with the epigenetic regulation of gene expression [279]; and the hepatitis C virus (HCV) and adenoviruses, which use proteins to disrupt epigenetic functions and alter global immune responses [280,281].

Finally, occupational characteristics have been studied as risk factors for several age-related diseases and are believed to influence the aging process. Andrasfay and Crimmins [282] found that manual labor and occupational physical activity might contribute to epigenetic age acceleration via their associations with socioeconomic status. In turn, work-related stress may act as a risk factor for epigenetic age acceleration by influencing health behaviors outside of work. However, conditions involving high work-related stress, high physical effort, and long working hours were not significantly linked to epigenetic aging.

## 4. The Role of the Gut Microbiome in Epigenetic Aging

Ghosh et al. [283] suggested that the GM is a contributory factor that modulates healthy aging and may be involved in several non-communicable diseases in all age groups. Individuals who age healthily have a distinct GM composition and diversity compared to young adults [284]. Research has revealed that the ratio of Bacillota/Bacteroidota phyla decreases with age, dropping from 10.9 in individuals aged 25–45 years old to 0.6 in those over 70 years old [285,286,287]. The age-related dysbiosis of the GM is primarily marked by a decline in microbial diversity, a reduction in beneficial bacteria, and an increase in the levels of potentially harmful microorganisms [288]. Additionally, the decreased production of beneficial metabolites such as short-chain fatty acids (SCFAs) may be linked to the pathophysiological changes and cognitive deterioration associated with aging [289,290]. Studies on healthy aging individuals have reported a reduction in various bacterial groups compared to the microbiome of young adults, including members of the phylum Actinomycetota and families such as *Bacteroidaceae*, *Lachnospiraceae*, *Oscillospiraceae*, and *Ruminococcaceae* [291]. The dominant genera found in these individuals include *Bacteroides*, *Bifidobacterium*, *Coprococcus*, *Dorea*, *Eubacterium*, *Faecalibacterium*, *Lactobacillus*, *Prevotella*, and *Roseburia* [292,293,294]. Conversely, age is associated with an increase in opportunistic pathogens and commensal bacteria linked to the release of pro-inflammatory cytokines, such as members of the families *Christensenellaceae*, *Barnesiellaceae*, and *Enterobacteriaceae* [291], as well as the genera *Akkermansia*, *Anaerotruncus*, *Barnesiella*, *Bilophila*, *Butyricimonas*, *Butyrivibrio*, *Clostridium*, *Coprobacillus*, *Desulfovibrio*, *Eggerthella*, *Flavonifractor*, *Klebsiella*, *Odoribacter*, *Oscillospira*, *Ruminococcus*, and *Streptococcus* [292,293,295].

Aging is linked to a gradual disruption of the physiological equilibrium between the host and the GM [296]. Nevertheless, it remains unclear whether or not these changes are driven by physiological alterations, age-associated neuroinflammation or inflammaging, diet, drug use, immunosenescence, compromised gut barrier function, or persistent health conditions [1,297,298,299,300]. Ghosh et al. [283] noted that the GM may serve as a potential modulator of aging and proposed that the microbial groups altered in healthy aging change in unhealthy aging due to factors such as fragility, cognitive disorders, cardiovascular and chronic kidney diseases, metabolic syndrome and obesity, reduced physical activity, and neurodegenerative diseases.

### 4.1. Gut Microbiome and Age-Related Epigenetic Changes

In addition to the nuclear genome, humans also carry mitochondrial DNA and the genomes of thousands of microorganisms that collectively form the human microbiome. Over 3.8 million microbial genes have been identified, representing a number 150 times greater than the total human genome, with 99% of these genes originating from bacteria [301]. Epigenetic modifications are present across all human genomes, which means that DNA methylation can also take place in mitochondrial and microbial DNA [302,303]. Mitochondrial DNA methylation has been shown to regulate various functions and may undergo alterations in certain cortical neurons with aging, as well as in specific neurodegenerative diseases [304,305].

DNA methylation serves as a fundamental mechanism of epigenetic regulation in bacteria, playing crucial roles in processes such as chromosome replication, cell cycle regulation, segregation, DNA repair, and transcription control. In many bacterial species, DNA methylation regulates reversible gene expression switching, known as phase variation, which leads to the generation of distinct phenotypic cell variants. The establishment of epigenetic marks enables bacterial populations to adapt to harsh or fluctuating environments and influences their interactions with eukaryotic hosts [306]. In this context, it has been proposed that bacterial methyltransferases contribute to clinically significant phenotypes, including virulence, host colonization, sporulation, biofilm formation, and phage defense [307,308,309]. Additionally, bacterial DNA methylation can play a protective role by safeguarding its own DNA through the restriction-modification system, which primarily involves endonucleases that target and degrade foreign, unmethylated DNA, such as that from bacteriophages [310].

Bacterial methyltransferases have been shown to play an important role in epigenetic regulation at the microbe level and are also key elements in microbe–host interactions. Certainly, secreted nucleomodulins, which are bacterial effectors that specifically target the nucleus of infected cells, have been demonstrated to directly alter the host’s epigenetic landscape. A subset of these nucleomodulins encodes methyltransferase activities that target both host DNA and histone proteins, resulting in significant transcriptional modifications within the host cell [311].

Gut bacteria may induce epigenetic changes in the host through infection processes [140]. For instance, infection with *H*. *pylori* has been shown to facilitate DNA methylation in the gastric mucosa [312], while *E*. *coli* (specifically uropathogenic UPEC strains) is capable of inducing DNA methylation in eukaryotic cells [313]. In addition, bacterial metabolites interacting with human cells have been reported to influence DNA methylation patterns, potentially contributing to neurological conditions [314,315,316]. Regarding neuronal cells, some of the age-related methylation alterations discussed previously have been correlated with an increased risk of various neurodegenerative diseases [317]. In summary, the GM plays a critical role in regulating brain cell functions through modifications in DNA methylation and histone patterns; however, the molecular mechanisms and signaling pathways driving this process are still not fully understood. Table 3 shows the GM dysbiosis related to unhealthy aging due to several disorders (modified from [295,318,319,320,321,322,323,324]).

### 4.2. Microbial Products and Host Epigenome

GM dysbiosis may lead to alterations in microbial metabolites, which can act as indirect regulators of the host’s epigenome. Intestinal microorganisms produce a wide range of small bioactive molecules, including SCFAs and other microbial byproducts such as β-hydroxybutyrate, folate, and other B vitamins, all of which play a significant role in physiological and epigenetic regulation [325]. SCFAs are exclusively produced through the microbial fermentation of dietary carbohydrates, and their levels are influenced by the microbial community composition [326]. These SCFAs are crucial inhibitors of histone deacetylases (HDACs) in the host, enzymes that remove acetyl groups from histones, thereby promoting chromatin condensation and silencing gene expression [325,327,328]. In addition, SCFA metabolism may increase cytoplasmic NAD^+^ concentrations and enhance sirtuin (deacetylase enzymes) activity by reducing the need to shuttle cytoplasmic NAD^+^ to the mitochondria, as compared to the demands of glucose metabolism [329].

SCFAs can also influence histone decrotonylation and acylation [330] and serve as donor substrates for HATs [331]. Furthermore, SCFAs can influence the activity of ten-eleven translocation methylcytosine dioxygenase (TET) enzymes, which are key players in DNA demethylation and generally promote gene transcription when targeted against gene promoters [332]. While butyrate and propionate act as inhibitors of sirtuins [333], acetate contributes to the pool of intermediate molecules that form acetyl-coenzyme A (CoA), the primary donor of acetyl groups for HATs, and it also inhibits HDACs, leading to enhanced histone acetylation and chromatin relaxation, as well as promoting a transcriptionally active chromatin state [334].

Alternative microbial metabolites, such as B vitamins and methionine, serve as donors of methyl and acetyl groups by producing S-adenosylmethionine (SAM), which acts as a substrate for DNMTs and HMTs [335]. The ratio of S-adenosylhomocysteine (SAH) to SAM modulates the overall methylation balance of the genome, influencing both DNA and histone methylation level [315]. Numerous studies have observed microbiota-driven alterations in genome-wide DNA methylation, also known as the methylome [336], and histone acetylation levels in intestinal epithelial cells [337]. Folate (vitamin B9) is primarily provided by the diet, but it can also be produced by intestinal microbiota like *Bifidobacterium bifidum*, *B*. *longum*, and *Limosilactobacillus reuteri* [338]. Most of the B vitamins, including biotin (vitamin B7), cobalamin (vitamin B12), niacin (vitamin B3), pantothenic acid (vitamin B5), and riboflavin (vitamin B2), are produced by the microbiota or ingested in the diet and are important cofactors of the folate cycle involved in epigenetic processes [339]. Riboflavin (vitamin B2) acts as a cofactor for methylenetetrahydrofolate reductase (MTHFR), a folate-dependent enzyme involved in one-carbon metabolism, which plays a role in DNA methylation [340]. Nicotinamide adenine dinucleotide (NAD), the active form of niacin, serves as a cofactor for NAD-dependent HDACs, which facilitate histone deacetylation [341]. Pantothenate is the primary acetyl group donor in the conversion of CoA to acetyl-CoA, and an adequate supply of acetyl-CoA supports acetyl-CoA-dependent histone acetylation [342]. There is a lack of evidence to confirm the direct involvement of vitamin B12 in epigenetic modifications. Ge et al. [343] demonstrated that the addition of cobalamin to human ileal epithelial cell cultures led to the enrichment of methyl donor SAM and accordingly increased the genome-wide CpG methylation, resulting in the differential methylations of genes involved in intestinal barrier function (MUC13) and cell proliferation (e.g., SHH). Table 4 shows the microbial metabolites, their producing bacteria, and their effects on the host epigenome (according to [344,345,346]).

Tricarboxylic acid (TCA) cycle intermediates, both microbial and host cell, are known to positively or negatively regulate histone methylation. For instance, alpha-ketoglutarate (α-KG) serves as a critical substrate for jumonji C histone demethylases (jmjC), while elevated levels of succinate and fumarate have been shown to inhibit the activity of jmjC demethylases. In turn, α-ketoglutarate is a co-substrate of TET dioxygenases, which are responsible for the demethylation processes of histones and DNA [347]. As for jmjC demethylases, elevated concentrations of fumarate and succinate can suppress TET enzymes, resulting in heightened levels of both histone and DNA methylation [348].

### 4.3. Gut Microbiome Dysbiosis and Epigenetic Regulation in Neurodegenerative Diseases

Neurodegeneration is a complex process leading to irreversible neuronal damage and death, and it is a common final stage in aging and various neurodegenerative diseases, all of which are characterized by neuronal loss that culminates in a state of dementia [40,349]. Alzheimer’s disease (AD), the most extensively studied neurodegenerative disorder, is marked by the buildup of extracellular amyloid beta (Aβ-42) plaques, which deprive neurons of essential nutrients and trophic factors, alongside intracellular neurofibrillary tangles (NFTs) that obstruct the axonal transport system, ultimately resulting in neurodegeneration [305]. NFTs, which are composed of hyperphosphorylated tau protein, and Aβ-42 plaques, which form due to the abnormal cleavage of amyloid precursor protein (APP) by β/γ-secretase enzymes, are key hallmarks of AD [37]. In addition to AD, other neurodegenerative diseases contributing to dementia in the elderly include Parkinson’s disease (PD), Huntington’s disease (HD), amyotrophic lateral sclerosis (ALS), and multiple sclerosis (MS) [291,350].

Several studies have been conducted to understand the molecular foundations of neurodegenerative processes [78], for example, a large-scale dysregulation of histone acetylation and chromatin remodeling due to tau pathology in the human prefrontal cortex [351], which is linked to memory decline and AD [352], lysine H3K27 acetylation (H3K27ac) in the entorhinal cortex, and differentially acetylated peaks that were enriched in disease-related biological pathways, including the genes involved in the progression of amyloid-β and tau pathology (e.g., *APP*, *PSEN1*, *PSEN2*, and *MAPT*) [353]. Altered histone acetylation has been observed not only in AD but also in other neurodegenerative disorders, including ALS [354], HD [355], PD [356], and MS [357].

The aggregation of α-synuclein is a defining characteristic of PD, with its spread beginning in the enteric nervous system, which governs gastrointestinal tract functions, before advancing to the CNS. In the CNS, α-synuclein aggregates, which form Lewy bodies, are linked to dopaminergic neuronal loss in the substantia nigra [358]. Since α-synuclein is transported from the gut to the brain via the vagus nerve, some researchers have suggested that vagotomy could serve as a potential therapeutic approach for PD [359,360]. This evidence implies that PD may originate in the gut and then progress to the brain, where δ-secretase may play a role in facilitating the formation of α-synuclein fibers [361]. Additionally, gastrointestinal dysfunction is considered one of the most prevalent non-motor symptoms of PD, impacting up to 80% of individuals affected by the disease [140].

A preclinical study using a mouse model of AD identified punctate alterations in DNA methylation, known as differentially methylated regions (DMRs), which were linked to specific brain areas [362]. The study also found that 21 gut bacterial species were significant predictors of some of these DMRs, along with certain behavioral outcomes in mice. Among the most significant DMRs were apolipoprotein E, associated with the *Muribaculaceae* family, and apolipoprotein C2, linked to the *Lachnospiraceae* family. Other DMRs were associated with AD-related genes such as ceramide kinase-like (*CerkI*) and glucagon-like peptide-2 receptor (*GLP2R*), both of which are involved in neurite function and spatial cognition, processes that are known to decline with aging. These DMRs showed a positive correlation with *Lachnoclostridium* and a negative correlation with *Muribaculaceae*. Above all, the findings revealed a connection between the presence of the *Lachnospiraceae* family in the GM and epigenetic modifications related to the hippocampal epigenetic landscape.

Only a few studies have described changes in the GM associated with PD. In a cohort of 64 Italian PD patients, the most significant microbiota changes were observed in a decrease in bacterial taxa associated with anti-inflammatory and neuroprotective effects. Specifically, there was a reduction in members of the *Lachnospiraceae* family and several genera, including *Butyrivibrio*, *Pseudobutyrivibrio*, *Coprococcus*, and *Blautia*. These microbiota changes were correlated with alterations in various classes of metabolites, such as lipids, vitamins, amino acids, and other organic compounds. This suggests a synergistic relationship between gut bacteria and their metabolites that contributes to altered homeostasis in PD. Furthermore, a reduction in SCFA-producing bacteria appeared to impact the metabolomic profile, affecting several metabolites with potential neuroprotective effects in the PD group [363]. In another study, Xie et al. [364] found that a decreased abundance of butyrate-producing bacteria, including *Roseburia*, *Romboutsia*, and *Prevotella*, was linked to epigenetic modifications in the leukocytes and neurons of patients with PD, as well as to the severity of their depressive symptoms.

## 5. Discussion

The GM can be considered as an epigenetic factor, influencing the host phenotype through both transient and potentially transgenerational effects [365]. Its capacity to mediate interactions between genes and the environment implies that the GM serves as a source of adaptive variation, contributing to phenotypic plasticity [20,366]. The close symbiotic relationship between host genetics and the GM is very ancient, as vertebrates co-evolved with their gut bacteria [367]. Divergence times also indicate that the chromosomal, mitochondrial, and GM genomes diversified together during hominin evolution [368,369].

The concept of the epigenome–microbiome axis establishes a bidirectional connection between the host and the GM, where the GM exerts control over the epigenome of the host. Additionally, the host exerts a complex influence on its microbiome, such as through histone and DNA modifications that affect the expression of immune genes, antimicrobial peptides, or gut barrier function, as well as through ncRNAs that likely impact microbial gene expression [22].

Several studies on human GM-induced changes in the host epigenome have elucidated potential mechanisms through which the epigenome–microbiome axis may operate, particularly in a pathogenic context [327,370,371]. However, the role of this phenomenon in aging is still under investigation. Diverse studies have observed GM-driven effects on genome-wide DNA methylation (i.e., the methylome) [336] and histone acetylation levels in intestinal epithelial cells [337]. Furthermore, RNA modifications and the regulation of ncRNAs have been suggested to play a role in the regulation of aging [6]. These epigenetic alterations can influence the transcriptional profile of the host, affecting various molecular and cellular responses and signaling pathways [372,373].

There is emerging interest in the diagnostic and therapeutic potential of the altered GM and epigenetic regulation in clinical practice. However, studies linking GM–epigenome correlations to aging diagnosis and treatment are still in the early stages, due to inconsistent findings regarding GM composition across different elderly populations. These variations are influenced by factors such as diet, culture, ethnicity, environmental differences, and dissimilarity in the methodologies used for analysis [291,374,375,376,377]. For example, the *Bacteroides* enterotype is most commonly found in Western countries with high-fat and protein diets, while the *Prevotella* enterotype is more prevalent in regions with high dietary fiber consumption [326].

This review highlights several key limitations related to current methodologies in microbiome research, particularly in the context of understanding the complex interactions between the GM and the epigenome of the host. Despite recent advances, the methodologies still face challenges in accurately capturing the full complexity of microbiome diversity, function, and its influence on host health, especially in specific populations like the elderly and those affected by neurodegenerative conditions. In this regard, advances in technology, such as next-generation meta-omics and high-throughput sequencing techniques (e.g., 16S ribosomal RNA microbial profiling, metabolomics, metatranscriptomics, shotgun metagenomics, and DNA microarrays), will potentially be able to enhance our understanding of the gut environment in elderly individuals and neurodegenerative patients through functional approaches. These innovations will help determine whether or not GM imbalances play a role in the early stages of dementia onset [378]. Additionally, biotechnological progress will provide significant opportunities for experimentally exploring the mechanisms driving the epigenome–microbiome axis. In recent years, the field of epigenetic engineering has advanced rapidly, particularly with the development of clustered regularly interspaced short palindromic repeat (CRISPR)-associated (Cas) protein systems [379]. Given the variability of diseases across different age groups, a multi-omics approach, combined with artificial intelligence and machine learning to analyze and integrate data, will be essential to gaining a deeper understanding of the gut–brain axis in neurological disorder pathogenesis. This approach will also help generate predictive models and facilitate a way for precise, personalized therapeutics [380]. Furthermore, while the majority of studies have predominantly focused on the bacterial component of the GM, the interactions between the epigenome of the host and other microbiota components, such as the virome, the mycobiome, and the microbiomes of different tissues [381,382,383,384], remain largely unexplored. This gap in research underscores the need for a more comprehensive approach that considers the complex and dynamic roles of viruses, fungi, and other microbial communities in shaping the epigenetic landscape of the host.

Several genome-wide association studies (GWASs) have been conducted to investigate the interplay between human genetic variation and GM composition, examining its potential influence on aging. As the field of genome–microbiome interactions remains in its early stages, metagenome-wide association studies (MGWASs) hold promise for further uncovering complex associations, aided by resources such as the Human Microbiome Project and advancements in bioinformatics tools. However, several key factors are often overlooked when studying the relationship between diet, host genome, epigenome, and GM in the context of disease and health. These factors include hygiene, disease history and current status, medication treatments, immune system functioning, age, smoking habits, lifestyle, overall health, and geographic location [385].

Cognitive stimulation, along with other interventions aimed at promoting healthy aging, could potentially act as epigenetic modulators by influencing gene expression related to brain health and neuroplasticity. However, the specific mechanisms by which these interventions might induce epigenetic changes remain unclear, and the duration of exposure required for these effects is not yet fully understood. While it is suggested that prolonged exposure to external factors like physical activity, which is often included in such programs, may induce epigenetic alterations, the pathways involved are complex and dependent on individual factors, including specific age range, genetic background, and overall health. In this respect, developing protocols that incorporate personalized lifestyle interventions aimed at promoting healthy aging could be highly beneficial for promoting longevity and quality of life among elderly populations. Nevertheless, such an initiative would first require the optimization of existing practices, focusing on identifying the key factors that make these programs more effective for older adults (e.g., frequency and duration of sessions, or the simultaneous combination of cognitive and physical exercises). Moreover, these programs should be standardized according to specific contexts, with an emphasis on personalization within those contexts, while overcoming the methodological limitations of current studies in the field, in order to obtain reliable results and enable the replication of effective practices [386,387].

On the other hand, the fermentation of dietary fibers in the gut alters the microbial ecosystem, leading to the production of metabolites such as SCFAs and also postbiotics. These metabolites have been shown to improve gut immune function, maintain blood–brain barrier integrity, and provide energy supply and pathogen defense [388]. Through these pathways, they may indirectly modulate epigenetic regulation, particularly in relation to inflammation and oxidative stress. In this regard, plant-based diets have been considered particularly beneficial due to their positive effects on the GM [291,388]. Thus, it could be hypothesized that these diets, by promoting a healthy GM, might play an important role in modulating epigenetic changes associated with aging and age-related diseases.

## 6. Conclusions

Extrinsic factors are potential inducers of epigenetic alterations via endocrine and immune pathways, which may have important implications for longevity. The GM affects brain cell activities through histone and DNA modifications and can therefore be considered as an epigenetic effector influencing host gene expression in aging, although studies on the molecular and signaling pathways underlying such interactions continue to be insufficient. On the other hand, the host may also be able to influence the GM by modulating the expression of immune-related genes, gut barrier function, and antimicrobial peptides, as well as via ncRNAs that likely affect microbial gene expression. Not much is known regarding the role of microbial metabolites on the host genome, and their influence on various age-related pathophysiological processes, including dementia and neurodegenerative diseases. In addition, while much of the research has concentrated on the bacterial aspect of the GM, the interactions between the host epigenome and the virome, mycobiome, or microbiomes of other tissues remain largely understudied. Ultimately, a comprehensive understanding of neuroscience, age-related brain disorders, and the human microbiome is expected to facilitate the way for strategies targeting the GM, offering new, safe, and effective therapeutic approaches for age-related conditions in the near future.

## Figures and Tables

**Table 1:** Chemical and xenobiotic influence in epigenetic changes of the nervous system.

Substances	Epigenetic Change	Effect	Reference
Heavy metals	DNA methylation	Synthesis of Aβ peptide	[155]
		Neurotoxicity	[156]
		Neurodegeneration and dementia	[157]
Manganese	DNA methylation (*PINK1* and *PARK2*)	Mitochondrial function	[158]
		Neurodegeneration	[159]
Lead	Hypermethylation in the hippocampus	Neurobehavioral and neurodevelopmental deficits	[160,161]
	Decreased expression levels of *PAX6* and *MSI1*	Shorter neuritas, reduced branching	[162]
		Promotion of neurodegenerative mechanisms	[163,164]
Methylmercury	Suppression of BDNF	Depression-like symptoms	[165]
	Suppress expression of *TH*	Neurodevelopmental effects	[166]
		Disruption of neuronal development	[167,168]
Arsenic	Hypoacetylation	Cognitive impairment	[169]
		Aberrant gene expression in brain	[170,171]
Bisphenol A	Disruption of *KCC2*	Detrimental neurodevelopment	[172]
		Regulation of chloride levels in neurons	[173]
	Change in mRNA of DNA methyltransferases	Behavioral changes	[174,175]
Polystyrene microplastic (PSMCs)	DNA methylation	Detrimental neurodevelopment	[176]
Polycyclic aromatic hydrocarbons (PAHs)	Increased DNA methylation of BDNF	Decrease in neurogenesis and neuronal survival	[177]
	Hypomethylation in *ZIC4* promoter region	Neural tube defects	[178]
Organophosphates and fluorophosphates pesticides	Deacetylation of histones in hippocampus	Chronic depression-like symptoms	[179]
	Histone hyperacetylation	Degeneration of dopaminergic neurons	[180]
Permethrin (insecticide)	DNA methylation	Transgenerational behavioral changes and neurodegeneration impairment	[181,182]

BDNF: Brain-derived neurotrophic factor; *KCC2*: K^+^/Cl^−^ cotransporter 2 gene; *MSI1*: Musashi RNA binding protein gene; *PARK2*: E3 ubiquitin ligase gene; *PAX6*: Paired box 6 gene; *PINK1*: Putative kinase 1 gene; *TH*: Tyrosine hydroxylase gene; *ZIC4*: Zic family member 4 gene.

**Table 2:** Influence of dietary components on the gut microbiome, epigenetic changes, and related-biological activities.

Food Components	Microbial Substrate	Microbial Metabolite Produced	Epigenetic Changes	Biological Activities
Carbohydrates	Non-starch	SCFAs (acetate, propionate, and butyrate)	Inhibition of HDAC activity	- Apoptosis - Inhibition of cell proliferation - Anticancer
	Resistant starch			
	Oligosaccharides			
Phytochemicals	Lignans	Ellagic acids	Inhibition of HAT activity	- Anti-inflammatory - Anticancer
	Flavonoids	Urolithins	Modulation of DNA methylation	
	Anthocyanins	Valerolactones	Modulation of histone modifications	
Fats	Polyunsaturated fatty acids	Linoleic acids	Induction of DNA methylation Induction of histone modifications	- Apoptosis - Anti-inflammatory - Anticancer
Fats	Undigested fats	Ursodeoxycholates	Modulation of genes at mRNA level	- DNA damage - Alteration in gene expression - Apoptosis
		Deoxycholates	Production of ROS	
Fats	Alcohol	Acetaldehyde	Inhibition of DNA repair system	- Carcinogenic activity
			Chromosomal damage	
			Alteration in gene expression	
Proteins	Amino acids (valine, leucine, and isoleucine)	SCFAs (acetate, propionate, and butyrate)	Inhibition of HDAC activity	- Apoptosis - Inhibition of cell proliferation - Anticancer
Proteins	Undigested amino acids (methionine, arginine, phenylamine, and tryptophan)	Polyamines	Alteration in cell growth	- Carcinogenic activity
		Phenols		
		Indoles		

SCFAs: Short-chain fatty acids; HDAC: Histone deacetyltransferase; HAT: Histone acetyltransferase; ROS: Reactive oxygen species.

**Table 3:** Gut microbiome dysbiosis related to unhealthy aging due to several disorders.

Disorders	Increased Abundance	Decreased Abundance
Fragility	Genera: *Anaerotruncus*, *Coprobacillus*, *Parabacteroides*, *Prevotella*, *Ruminococcus* Family: *Atopobiaceae*	Alfa-diversity Genera: *Azospira*, *Coprococcus*, *Eubacterium*, *Faecalibacterium*, *Gemella* Families: *Barnesiellaceae*, *Christensenellaceace*
Reduced physical activity	Genera: *Anaerotruncus*, *Bilophila*, *Coprobacillus*, *Eggerthella*, *Megasphaera*	Genera: *Cetobacterium*, *Faecalibacterium*, *Prevotella* Family: *Lachnospiraceae*
Obesity and metabolic syndrome	Genera: *Bifidobacterium*, *Collinsella*, *Paraprevotella*	Genera: *Akkermansia*, *Faecalibacterium*, *Prevotella*
Cardiometabolic and chronic kidney disease	Genera: *Lactobacillus* Species: *Clostridioides difficile*	Genera: *Faecalibacterium*, *Oscillospira*, *Prevotella*, *Roseburia*
Neurodegenerative diseases	Pathobionts	Genera: *Adlercreutzia*, *Bifidobacterium*, *Butyrivibrio*, *Odoribacter*, *Prevotella*, *Victivallis*
Cognitive decline	Genera: *Blautia*, *Lactobacillus*, *Ruminococcus*	Alfa-diversity Genera: *Akkermansia*, *Barnesiella*, *Butyricimonas*, *Lachnospira*, *Lentisphaera*, *Odoribacter*, *Prevotella* Families: *Christensenellaceace*

**Table 4:** Microbial metabolites, role in epigenetic mechanisms, and producing bacteria.

Microbial Metabolite	Role in Epigenetic Mechanisms	Producing Bacteria
SCFAs:		
Acetate	Inhibitor of HDACs Increase HATs	*Acetobacter*, *Acetogenium*, *Akkermansia*, *Bacteroides*, *Bifidobacterium*, *Blautia*, *Clostridium*, *Eubacterium*, *Prevotella*, *Ruminococcus*, *Streptococcus*
Propionate	Inhibitor of sirtuins deacetylase enzymes	*Anaerostipes*, *Bacteroides*, *Coprococcus*, *Dialister*, *Eubacterium*, *Faecalibacterium*, *Megasphaera*, *Ruminococcus*, *Salmonella*, *Veillonella*
Butyrate	Inhibitor of sirtuins	*Acidaminococcus*, *Anaerostipes*, *Bifidobacterium*, *Clostridium*, *Coprococcus*, *Eubacterium*, *Faecalibacterium*, *Fusobacterium*, *Peptostreptococcus*, *Roseburia*
Vitamins B group:		
B2 (riboflavin)	Cofactor of MTHFR involved in DNA methylation	Members of the phyla Pseudomonadota and Fusobacteriota
B3 (niacin)	Cofactor of NAD-dependent HDACs	*Bacteroides fragilis*, *Prevotella copri*, *Ruminococcus lactaris*
B5 (pantothenic acid)	Donor of acetyl groups involved in acetyl-CoA-dependent histone methylation	*Corynebacterium glutamicum*, *Escherichia coli*, *Salmonella typhimurium*
B7 (biotin)	Involved in DNA methylation	*Bacteroides fragilkis*, *Campylobacter coli*, *Fusobacterium varium*, *Prevotella copri*
B9 (folate)	Regulation of methylation of DNA and histone	*Bifidobacterium* spp., *Limosilactobacillus reuteri*
B12 (cobalamin)	Donor of acetyl groups, reconfigure human epigenome and mitochondrial genes	*Bacillus megaterium*, *Bifidobacterium* spp., *Faecalibacterium prausnitzii*, *Fusobacterium varium*, *Propionibacterium freudenreichii*, *Pseudomonas denitrificans*, *Ruminococcus lactaris*

SCFAs: Short-chain fatty acids; HDACs: Histone deacetyltransferases; HATs: Histone acetyltransferases; MTHFR: Methylentetrahydrofolate reductase; NAD: Nicotinamide adenine dinucleotide.
